# T346Hunter: A Novel Web-Based Tool for the Prediction of Type III, Type IV and Type VI Secretion Systems in Bacterial Genomes

**DOI:** 10.1371/journal.pone.0119317

**Published:** 2015-04-13

**Authors:** Pedro Manuel Martínez-García, Cayo Ramos, Pablo Rodríguez-Palenzuela

**Affiliations:** 1 Área de Genética, Facultad de Ciencias, Instituto de Hortofruticultura Subtropical y Mediterránea 'La Mayora', Universidad de Málaga, Consejo Superior de Investigaciones Científicas (IHSM-UMA-CSIC), Málaga, E-29071, Spain; 2 Centro de Biotecnología y Genómica de Plantas (CBGP), Universidad Politécnica de Madrid-Instituto Nacional de Investigación y Tecnología Agraria y Alimentaria, Parque Científico y Tecnológico de la Universidad Politécnica de Madrid, Campus de Montegancedo, Pozuelo de Alarcón, Madrid, 28223, Spain; 3 Departamento de Biotecnología, Escuela Técnica Superior de Ingenieros Agrónomos, Universidad Politécnica de Madrid, Avenida Complutense 3, Madrid, 28040, Spain; University of the West of England, UNITED KINGDOM

## Abstract

T346Hunter (Type Three, Four and Six secretion system Hunter) is a web-based tool for the identification and localisation of type III, type IV and type VI secretion systems (T3SS, T4SS and T6SS, respectively) clusters in bacterial genomes. Non-flagellar T3SS (NF-T3SS) and T6SS are complex molecular machines that deliver effector proteins from bacterial cells into the environment or into other eukaryotic or prokaryotic cells, with significant implications for pathogenesis of the strains encoding them. Meanwhile, T4SS is a more functionally diverse system, which is involved in not only effector translocation but also conjugation and DNA uptake/release. Development of control strategies against bacterial-mediated diseases requires genomic identification of the virulence arsenal of pathogenic bacteria, with T3SS, T4SS and T6SS being major determinants in this regard. Therefore, computational methods for systematic identification of these specialised machines are of particular interest. With the aim of facilitating this task, T346Hunter provides a user-friendly web-based tool for the prediction of T3SS, T4SS and T6SS clusters in newly sequenced bacterial genomes. After inspection of the available scientific literature, we constructed a database of hidden Markov model (HMM) protein profiles and sequences representing the various components of T3SS, T4SS and T6SS. T346Hunter performs searches of such a database against user-supplied bacterial sequences and localises enriched regions in any of these three types of secretion systems. Moreover, through the T346Hunter server, users can visualise the predicted clusters obtained for approximately 1700 bacterial chromosomes and plasmids. T346Hunter offers great help to researchers in advancing their understanding of the biological mechanisms in which these sophisticated molecular machines are involved. T346Hunter is freely available at http://bacterial-virulence-factors.cbgp.upm.es/T346Hunter.

## Introduction

The secretion of large molecules across the cell envelope is an essential bacterial mechanism involved in their survival and adaptation to diverse environments. Proteins are transported from the bacterial cell to the environment or directly into eukaryotic or prokaryotic cells [1,2]. The general secretory pathway (Sec) and the two-arginine (Tat) translocation pathway, which are universal machineries shared by bacteria, archaea and eukaryotes [3], are sufficient for protein secretion in Gram-positive bacteria. Meanwhile, in Gram-negative didermic bacteria, these two pathways translocate proteins into the periplasm but not across the outer membrane (OM). This second membrane system serves as a protective structure against antibiotics and antimicrobial host compounds and enables the colonisation of host environments. However, it also presents an impediment to protein secretion, and throughout evolution, Gram-negative bacteria have developed sophisticated mechanisms for the translocation of proteins across the cell envelope. Similarly, Gram-positive bacteria with a cell wall heavily modified by lipids, such as mycobacteria, have also evolved refined machineries for protein secretion [4]. So far, seven general classes of secretion systems have been identified, numbered T1SS to T7SS [3]. All these systems play a crucial role in the interaction of bacteria with the environment, particularly during the relationships established with eukaryotic host cells.

In terms of pathogenicity, secretion systems represent major virulence determinants for bacteria harbouring them. A broad range of secretion systems has been described for plant, animal, human and fish pathogens [3,5]. By means of secreted enzymes such as proteases, lipases and pectate lyases, bacteria are able to degrade eukaryotic cell wall components and metabolise host polymers by decomposing them. These enzymes are exported to the environment, and their secretion is executed mainly by T1SS, T2SS and T5SS [2]. On the other hand, effector proteins are injected into host cells by T3SS, T4SS and T6SS [6,7,8]. Effectors have the ability to produce physiological changes in the host, fulfilling essential functions during the interaction between bacteria and eukaryotes. T3SS effectors secreted by *Salmonella enterica*, a pathogen that causes gastroenteritis and typhoid fever in humans, help bacteria to modulate host immune signals [9]. VirB effectors delivered by the T4SS VirB system of *Brucella* contribute to the intracellular growth of this pathogen, as well as to its persistence in the livers of mice [10]. Meanwhile, effector protein delivery via T6SS has been shown to provoke actin cytoskeleton disruption and apoptosis in HeLa cells [11]. Toxins, which are secreted by all secretion systems, help pathogenic bacteria to promote infection by damaging host tissues and by modulating the host immune response [12]. Such is the case for the cholera toxin, secreted by *Vibrio cholerae* via the T2SS [13], and coronatine, a *Pseudomonas syringae* T3SS-secreted phytotoxin [14]. *Agrobacterium tumefaciens* promotes tumour formation in plants by transferring cytokinin- and auxin-coding genes to the plant from the T-DNA plasmid, which also encodes a T4SS required for its transfer [15].

Current research on secretion systems of pathogenic bacteria is targeted at identifying such systems in bacterial genomes and at characterising the functions of their secreted effectors, with a special focus on T3SS, T4SS and T6SS. There exists a wide variety of each of these systems and the effectors they deliver. Distribution of effector proteins varies both among different species and among different strains of the same species, and strains isolated from diverse locations may have significantly divergent effector repertoires [6]. Therefore, the design of new control strategies against bacterial pathogens first requires the identification of T3SS, T4SS and T6SS, and of their secreted effectors [16]. Functional studies will then allow a deeper understanding of the molecular mechanisms of action of these secretion systems and their targets in eukaryotic cells.

Due to the rapid progression of advances in genome sequencing and with plunging costs, a large amount of genomic data is being generated every day. Bacteriologists routinely make use of high-throughput sequencing technologies to obtain the whole genome sequences of strains of interest, requiring computational methods for automatic identification of bacterial virulence machineries. Nonetheless, few web-based applications have been developed to predict secretion systems components. SSPred is a web server based on support vector machine (SVM) for the prediction of proteins involved in bacterial secretion [17]. It takes a set of amino acid sequences as input and classifies it into T1SS, T2SS, T3SS, T4SS or Sec secretion system. However, a maximum of four amino acid sequences can be submitted, and genome-wide analyses cannot be performed. T3DB is a T3SS related database that provides a tool for the identification of T3SS genes given user-supplied genomic sequences [18]. Again, genome-wide searches are not supported, and sequences need to be supplied one by one. Guglielmini *et al*. [19] built the CONJscan-T4SSscan web server, which uses hidden Markov model (HMM) profiles to scan a set of protein sequences for T4SS components. Along similar lines, Abby and Rocha [20] implemented a web server, called T3SSscan-FLAGscan, for the identification of T3SS components. The latter makes use of HMM profiles to not only identify T3SS components but also discriminate between flagellar and non-flagellar ones, given a set of protein sequences. To our knowledge, no specific tool is available to predict T6SS components, and users typically rely on general annotation tools for their identification [21,22]

One limitation of the above servers is that each input sequence is analysed individually, which makes them not suitable for the localisation of genomic clusters. Such a feature is of special interest given that secretion systems components are typically encoded within virulence-associated plasmids or pathogenicity islands. Besides, most of those tools either generate raw outputs from BLAST [23] (T3DB) and HMMER [24] (T3SSscan-FLAGscan) or just produce a shallowly informative output of the predictions (SSPred). Only CONJscan-T4SSscan presents the results in a tab-delimited schema, which is certainly a more helpful format, but still requires some user manipulation. None of the before drawbacks is found in the annotation tool provided by SecReT4, an open-access web database of information on the T4SS [25]. A modest limitation of this tool is that it only accepts one input sequence, being not suitable for the analysis of draft genomes.

In this context, we developed T346Hunter (Type Three, Four and Six secretion system Hunter), a novel web-based tool designed to facilitate the identification of T3SS, T4SS and T6SS encoded in newly sequenced bacterial genomes. T346Hunter makes use of a database of HMM profiles and protein sequences to automatically annotate and localise T3SS, T4SS and T6SS in user-supplied bacterial genomes. By exploring the available scientific literature, we constructed a database of protein components that captures the diversity of these three types of secretion systems. Once the database search is performed and the secretion systems clusters have been localised, the system presents the results in a comprehensive and user-friendly formatted document, which can be accessed online or downloaded. Furthermore, T346Hunter accepts submissions of both complete and unfinished genomes. We think T346Hunter represents a valuable tool for researchers to help further their understanding of secretion systems in the context of pathogenesis.

## Material and Methods

### Protein sequences and profiles

Sequence profiles of secretion systems components were generated by selecting orthologues of each component in order to capture the diversity of the T3SS, T4SS and T6SS. Following the approach described by Abby and Rocha [20], we selected protein sequences corresponding to the components of the flagellar and non-flagellar T3SS (NF-T3SS). Meanwhile, to build profiles that represent the variety of T4SS, protein sequences of the components of 18 archetypal T4SS [25] were also selected. In both cases, sequences of each component were extracted from a set of model organisms representative of the diversity of all these types of systems. We based the construction of T6SS components profile on a previously reported list of coding sequences belonging to several bacterial genomes; sequences that were found to be orthologues of the components of the first described T6SS, namely, *V*. *cholerae*, *Pseudomonas aeruginosa* and *Burkholderia mallei* [26]. The sequences of these orthologues were also included in our set of protein families. All these sequences were downloaded from the NCBI website and selected based on their RefSeq genome annotation. Then, sequences corresponding to each component were aligned with Muscle [27] and manually adjusted with Seaview [28]. Finally, protein profiles were built with HMMER3 [24]. When fewer than five representative model organisms were found to code for a given component, no profile was built; instead, files were generated in multi-FASTA format.

We extended the above set by collecting protein sequences from AtlasT4SS [29] and proceeding in the same way as above to generate profiles of orthologue clusters described in that database. Secretion systems loci identified in this study were manually screened, and additional profiles were incorporated based on the RefSeq genome annotation. Consequently, our final dataset comprises sequence information for a total of 364 components of the T3SS, T4SS and T6SS (65, 449 and 20, respectively). Further information about each of the component profiles can be found in S1 Table.

### Identification of secretion systems clusters

T346Hunter performs BLASTp [23] and HMMER3 [24] searches of the protein sequences and profiles described above against user-supplied genomic sequences. Regions containing homologous genes (Evalue < = 0.0005) of at least 4 different core components of T3SS, T4SS or T6SS and spanning up to 70 kb are retained and included in the output report. We consider core components of T3SS, T4SS and T6SS as described by Abby and Rocha [20], Bi *et al*. [25] and Shrivastava and Mande [26], respectively (see S1 Table for details).

### Implementation

T346Hunter runs on a Linux platform with an Apache web server. The web interface was implemented using HTML and CSS, and data pipelines were developed using PHP, Perl, R and shell scripts. Circular genome images are generated using circos [30], and gene maps are produced using the R package genoPlotR [31]. Open reading frame predictions are generated with Glimmer v3.02 [32].

## Results and Discussion

### Using T346Hunter

**Fig 1:**
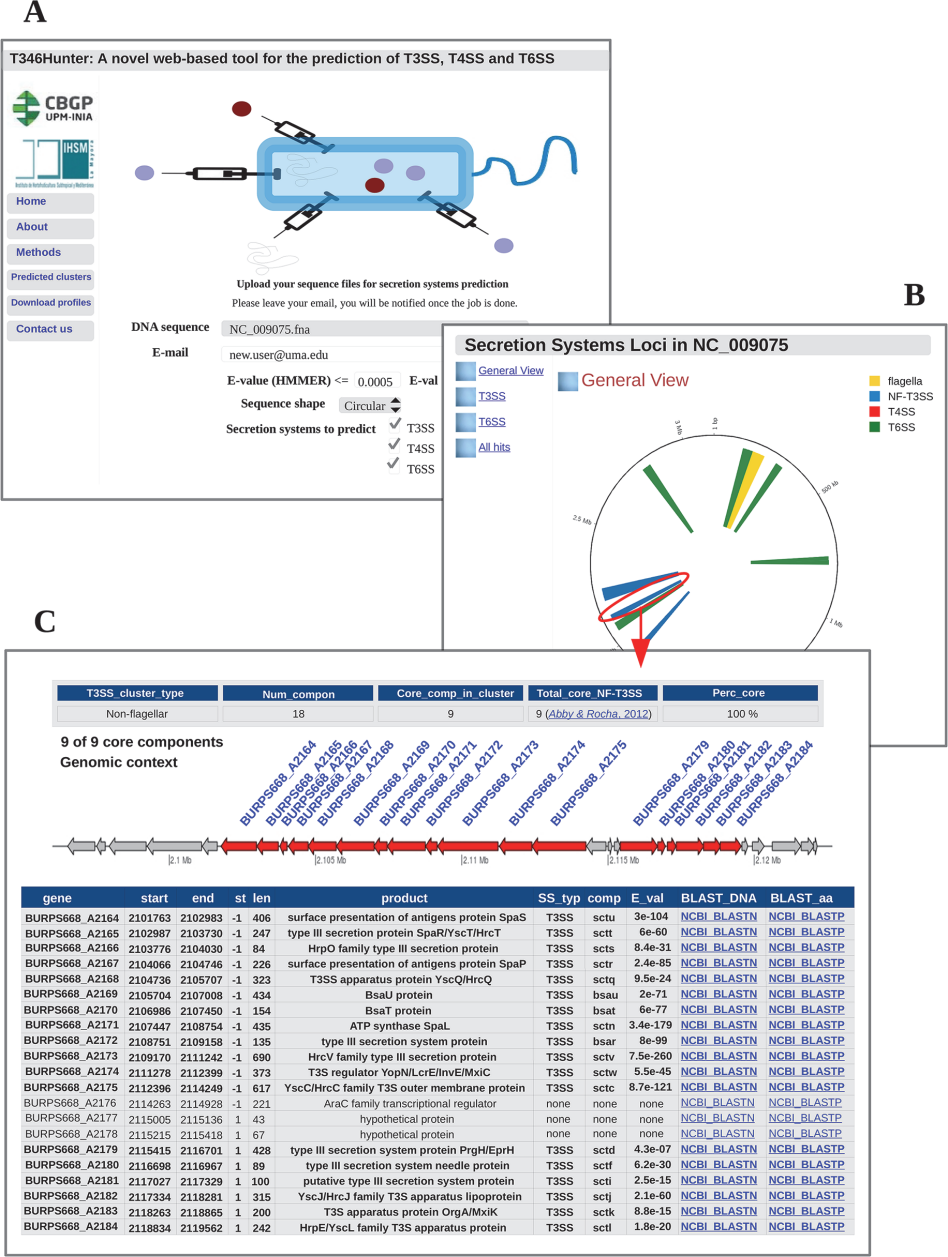
Example of execution of T346Hunter using the sequence of *B*. *pseudomallei* 668 chromosome 2 as input. A. Web interface of T346Hunter. A sequence file together with a hypothetical email address are shown as selected to be uploaded into the server. B. Genome-wide graphical view showing the predicted secretion systems of *B*. *pseudomallei* 668 chromosome 2. C. Genomic representation of one of the three NF-T3SS clusters identified, including a graphical gene map and a tabulated gene list with detailed information of each component. Hyperlinks to NCBI for direct execution of BLASTn and BLASTp against the non-redundant nucleotide and protein databases are provided for each gene within the loci. Some other relevant information is also included, such as the percentage of core components found in the cluster and PubMed hyperlinks to the studies we have based our methods on to build the component profiles found in such a cluster.

T346Hunter provides a simple and user-friendly web interface (Fig. 1A) for the search of T3SS, T4SS and T6SS in user-supplied genomic sequences. A user can upload the sequence of interest at http://bacterial-virulence-factors.cbgp.upm.es/T346Hunter in two ways. First, the user can provide raw DNA sequences in FASTA format, in which case the system makes use of Glimmer v3.02 [32] to predict coding regions. When more than one sequence is submitted, T346Hunter interprets the set of sequences as a draft genome (S1 Fig.). Alternatively, the user can upload an NCBI-formatted sequence. In such a case, three types of data have to be uploaded to the server, including DNA sequence, protein sequences and the location of protein-coding genes. The format of the data should be similar to that used by the NCBI Genome FTP Server (*fna*, *faa* and *ptt* files). Users are encouraged to submit their sequences in this format, since T346Hunter does not take phylogeny into account to predict coding regions, leading to a potential decrease in the quality of gene calls. Besides the input sequence, several parameters are provided for the user to configure the search, including HMMER3 [24] and BLASTp [23] E-value thresholds, secretion systems to be predicted and sequence shape (circular or linear). Once the prediction is completed, secretion systems loci are displayed in an intuitive HTML document containing tabulated and graphical information of the regions, along with a whole-genome graphical view. A tab-delimited summary of the gene-by-gene search, which can be easily incorporated into data pipelines, is also provided. These results are stored on the T346Hunter server for a week.

### T346Hunter output

Here, the result of the execution of T346Hunter on the sequence of *B*. *pseudomallei* strain 668 is shown as an example (Fig. 1). The genome of *B*. *pseudomallei* is typically comprised of two circular chromosomes, which encode several secretion systems [33,34,35]. *B*. *pseudomallei* is an aerobic, Gram-negative bacterium that infects humans, animals and even plants. It is the causative agent of melioidosis, an often-fatal disease that is endemic to Southeast Asia and Northern Australia, and whose infection can take place by ingestion, inhalation and skin abrasion. There is no vaccine available to protect against this pathogen, which is also highly resistant to antibiotics. All this makes it a potential organism to be used as a bioterrorism agent [36], and a growing interest on its virulence mechanisms has recently emerged. Consequently, hundreds of genome sequences are nowadays available for B. pseudomallei [37], and the role of T3SS and T6SS in the virulence of this species has been previously reported [34,35]. Fig. 1B shows the whole-genome graphical overview generated by T346Hunter displaying the predicted secretion systems clusters for B. *pseudomallei* 668 chromosome 2 (NCBI Refseq NC_009075). The system localises three clusters of NF-T3SS, one cluster of flagellar T3SS and five clusters of T6SS. These predictions are consistent with previously reported *in silico* analyses [20,38]. The bsa NF-T3SS of B. *pseudomallei* has been shown to be an important part of the virulence armoury of this strain [39]. Fig. 1C shows the output generated by T346Hunter containing detailed information of such cluster, including a tabulated output and a gene map graphic representing its genomic context.

### Core components

The sets of core components used for T3SS, T4SS and T6SS were as described by Abby and Rocha [20], Bi et al. [25] and Shrivastava and Mande [26], respectively. However, there is no consensus on the definition of core in terms of secretion systems components. As long as we understand, “core” is the minimum set of components experimentally proven to be necessary for a secretion system to be functional. That appears to be the meaning used by Abby and Rocha [20] and Shrivastava and Mande [26] when they suggest a set of T3SS core components and T6SS components of major requirement, respectively. On the other hand, Bi *et al*. [25] do not explicitly describe minimum required sets of components, but suggest a list of core components for each of the 18 T4SS they collect in their database. It is not clear though whether such proteins are indispensable for these T4SS to be functional. For instance, the trb T4SS encoded by A. *tumefaciens* C58 [40] lacks TrbN, which belongs to the above core list. Given this controversy, T346Hunter makes no discrimination regarding the different uses of the core set, leaving to the user the role of interpreting the results. We chose 4 as the minimum threshold of core components after trying different values and manually screening the predicted secretion systems, since it offers a trade-off between false positives and false negatives. Again, it is the user who has to sift through the predictions.

### Identification of T3SS, T4SS and T6SS gene clusters in sequenced bacterial genomes

Complete bacterial genomic sequences of 2997 chromosomes and 2164 plasmids sequenced available as of 14 February 2014 were downloaded from GenBank Refseq. T346Hunter was executed on these sequences and localised clusters enriched in either NF-T3SS, T4SS or T6SS components. In total, 2,814 clusters were identified (512 NF-T3SS, 1,466 T4SS and 836 T6SS) across 1,121 organisms. Predicted clusters are summarised in S2 Table and can be queried at http://bacterial-virulence-factors.cbgp.upm.es/T346Hunter#predicted_ref. Sequences with negative predictions are listed in S3 Table.

### Comparison with currently available tools

In order to validate the performance of T346Hunter, systematic comparisons with other available applications for secretion systems prediction would certainly be the best choice. By crossing our predictions with loci identified by other servers one could have a measure of the relative accuracy of our tool. But, in practise, this is not straightforward to carry out. On the one hand, predicted data are not always available in a format that are ready to be systematically analysed. Servers usually provide their data in a way that either they have to be queried using some kind of criteria (e.g. strain name) or they are just embedded in the webpage. On the other hand, few servers are available to predict secretion systems clusters as such. To our knowledge, only SecReT4 [25] provides a specific tool for genomic localisation of T4SS clusters. Despite these difficulties, and given the need for assessing the accuracy of our predictions relative to others' work, we attempted to accomplish comparisons either by systematic processing, when possible, or by manual inspection, when not.

First, we aimed to compare our predictions of T3SS with those of T3SSscan-FLAGscan [20]. Such server does not localise T3SS clusters in user-submitted sequences, but does keep a repository of predicted NF-T3SS loci that can be accessed using different features. Therefore, we randomly queried clusters for 100 genomic sequences and manually compared them with our predictions. Since negative predictions are not provided in the server, this selection was restricted to positive predictions. We found that T346Hunter predicted any NF-T3SS cluster in the 100 sequences examined. More precisely, T346Hunter and T3SSscan-FLAGscan identified the same number of clusters in 95 sequences (S4 Table). For each of the resting five sequences, the number of predicted clusters just differs in one. This difference is probably explained by the different searching criteria used by both methods, particularly in defining contiguous genes within a cluster and setting the minimum required number of core components. In total, 125 clusters were identified by T3SSscan-FLAGscan and 128 by T346Hunter.

To test how T346Hunter performs in predicting T4SS, we compared it with SecReT4 [25]. This server provides a summary of T4SS predictions on a number of sequences by means of a table in the webpage. We could, then, proceed to systematically compare the two methods. Again, such a comparison was necessarily restricted to positive predictions. In total, 387 sequences were examined and all of which were found to contain at least one T4SS cluster. Of 387 such replicons, 324 (∼84%) were predicted by both methods to contain the same number of T4SS (S5 Table). In this case, differences in searching criteria may have had a stronger impact in the predictions. SecReT4, for instance, does not restrict T4SS genes to be located in a specific cluster. In contrast, T346Hunter identifies a cluster whenever orthologues of 4 core components are found in a window of up to 70 kb. Despite such divergent parametrisation, 53 out of the 63 sequences with discordant predictions (∼84%) only differ in one cluster. Accounting for the 387 sequences analysed, SecReT4 and T346Hunter identified 522 and 595 T4SS, respectively.

We went ahead to inspect the accuracy of our T6SS predictions. As a specific server for the prediction of T6SS is not available, tool-by-tool comparison was precluded in this case. However, systematic localisation of T6SS loci has been previously performed [26,38,41], reporting data we could use to compare our predictions with. We focused on Boyer *et al*. [38], which completed the widest analysis. Since no readily processable summary of the predictions was provided, comparisons had to be manually performed. Among the 100 sequences reported with positive predictions, T346Hunter identified at least one T6SS in 98 of them. Furthermore, both approaches predicted the same number of T6SS clusters in 93 sequences (S6 Table). Such a subtle difference is explained by the requirement of 4 core components in a cluster imposed by T346Hunter, which was not applied by Boyer *et al*. [38]. Nonetheless, predictions on the resting 7 sequences of both approaches differed in only one cluster. Summing up all identified clusters in the 100 sequences examined, T346Hunter and Boyer *et al*. [38] predicted 170 and 175, respectively.

Regarding general annotation engines, some of the most widely used tools are the NCBI Prokaryotic Genome Annotation Pipeline [21] and the RAST server system [22]. In the last few years, RAST has become particularly popular and is now frequently used to rapidly annotate bacterial genomes against its comprehensively curated subsystem database. Due to its constant growth, RAST automated annotations are nowadays of a great quality, having reached a high degree of specificity. Indeed, T3SS, T4SS and T6SS are included among the subsystems collection of RAST, and thorough reports of related genes are provided within general annotations. Such reports include visual and tabular information of the corresponding genomic clusters, thus offering an exhaustive output. However, bacterial strains that has not been incorporated into RAST database are reported with no subsystems, and users need to manually inspect individual features to infer the existence of secretion systems clusters. This makes RAST subsystems search dependent on its database of bacterial isolates, and makes it particularly not suitable for the analysis of newly characterised bacteria. Furthermore, when subsystems are reported, the number of secretion systems clusters are not directly shown in the output and it rather needs to be derived from reported tables. Besides, no information regarding core components is provided, and some conjugal T4SS are not categorised as such. Therefore, even though RAST performs quite well in detecting genes encoding secretion systems when compared to other general annotation tools, its annotations lack some relevant information on T3SS, T4SS and T6SS, and do not directly offer the whole picture of the underlying genomic clusters.

## Conclusion

The development of web-based tools for the prediction of virulence factors is crucial for allowing researchers to identify the bacterial pathogenic arsenal. Here, we present T346Hunter, an online tool for annotation and localisation of secretion systems clusters in sequenced bacterial genomes. Because they are distinctive features of pathogenesis, T346Hunter searches for T3SS, T4SS and T6SS, whose identification is of particular interest in the development of strategies against bacterial-mediated diseases. The server will be continuously updated as new experimental and bioinformatics information on secretion systems becomes available. We believe T346Hunter will help researchers uncover the mechanisms of bacterial secretion as a virulence trait.

## Supporting Information

S1 Fig Overview of T346Hunter prediction workflow. (PDF)

S1 Table Protein profiles and sequences used in this study. (DOC)

S2 Table Summary of predicted clusters. (XLS)

S3 Table List of complete bacterial genomic sequences and plasmids not found to encode NF-T3SS, T4SS or T6SS. (XLS)

S4 Table Summary of NF-T3SS clusters predicted by T3SSscan-FLAGscan and T346Hunter on 100 sequences. (XLS)

S5 Table Summary of T4SS clusters predicted by SecReT4 and T346Hunter on 387 sequences. (XLS)

S6 Table Summary of T6SS clusters identified in Boyer et al. [38] and by T346Hunter on 100 sequences. (XLS)

S7 Table Predictions of secretion systems clusters not fulfilling the restriction of 4 core components. (XLS)

S1 Data Hidden Markov Model profiles and sequences used by T346Hunter. (ZIP)
