# Supplementary material for: T346Hunter: A Novel Web-Based Tool for the Prediction of Type III, Type IV and Type VI Secretion Systems in Bacterial Genomes
Source: PLoS One. 2015 Apr 13;10(4):e0119317. doi: 10.1371/journal.pone.0119317 (PMC4395097; doi:10.1371/journal.pone.0119317)
Supplement: S1 Fig — (PDF) [file pone.0119317.s001.pdf]

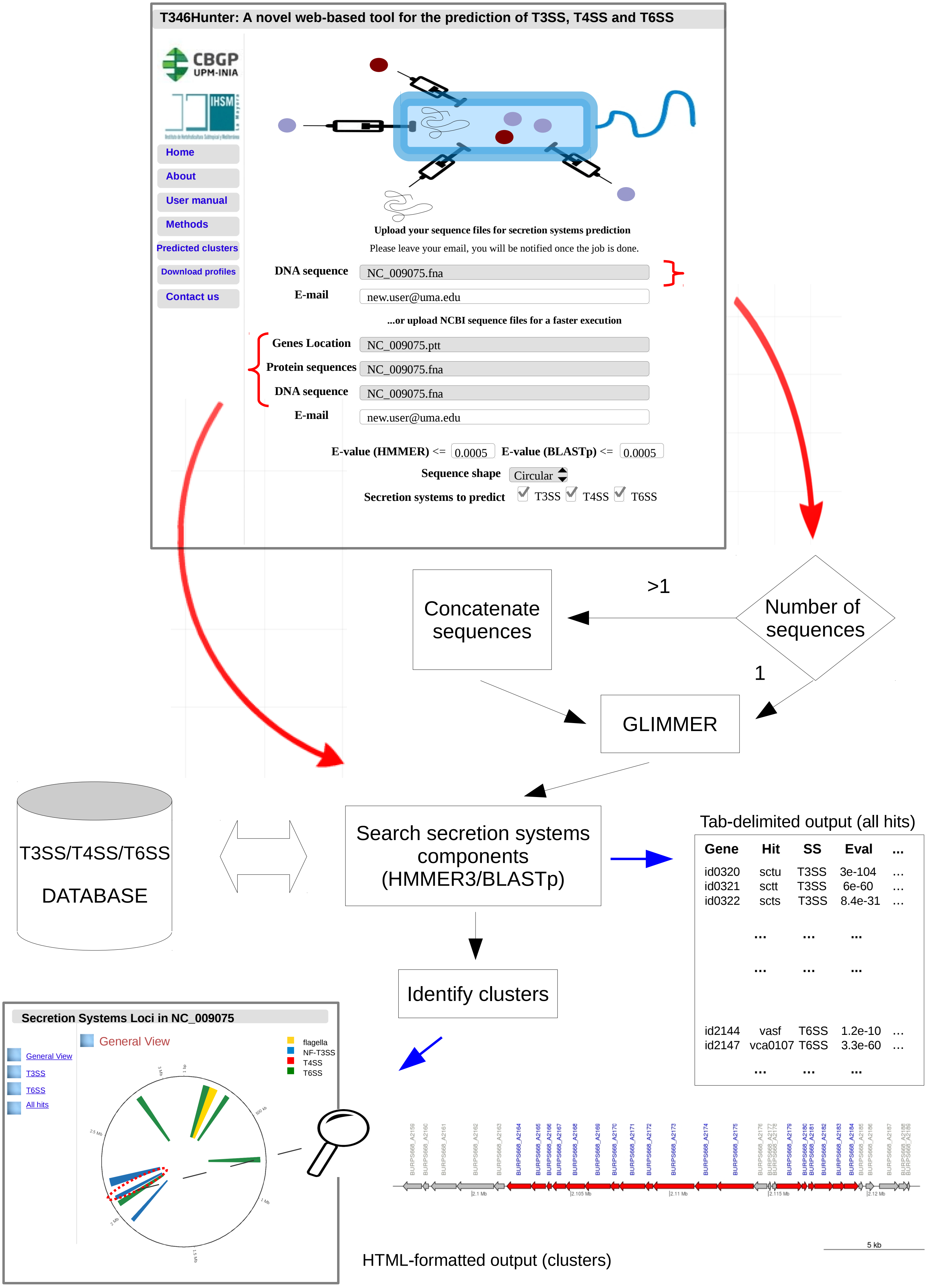

T346Hunter: A novel web-based tool for the prediction of T3SS, T4SS and T6SS
CBGP
UPM-INIA
IHSM
Home
About
User manual
Methods
Predicted clusters
Download profiles
Contact us
Upload your sequence files for secretion systems prediction
Please leave your email, you will be notified once the job is done.
DNA sequence
NC_009075.fna
E-mail
new.user@uma.edu
...or upload NCBI sequence files for a faster execution
Genes Location
NC_009075.ptt
Protein sequences
NC_009075.fna
DNA sequence
NC_009075.fna
E-mail
new.user@uma.edu
E-value (HMMER) <=
0.0005
E-value (BLASTp) <=
0.0005
Sequence shape
Circular
Secretion systems to predict
T3SS
T4SS
T6SS
Number of sequences
>1
1
Concatenate sequences
GLIMMER
T3SS/T4SS/T6SS DATABASE
Search secretion systems components (HMMER3/BLASTp)
Tab-delimited output (all hits)
Gene Hit SS Eval ...
id0320 sctu T3SS 3e-104 …
id0321 sctt T3SS 6e-60 …
id0322 scts T3SS 8.4e-31 …
id2144 vasf T6SS 1.2e-10 …
id2147 vca0107 T6SS 3.3e-60 …
Identify clusters
Secretion Systems Loci in NC_009075
General View
General View
T3SS
T6SS
All hits
flagella
NF-T3SS
T4SS
T6SS
5 kb
HTML-formatted output (clusters)
