# Supplementary material for: T346Hunter: A Novel Web-Based Tool for the Prediction of Type III, Type IV and Type VI Secretion Systems in Bacterial Genomes
Source: PLoS One. 2015 Apr 13;10(4):e0119317. doi: 10.1371/journal.pone.0119317 (PMC4395097; doi:10.1371/journal.pone.0119317)
Supplement: Supplementary file 2 [file pone.0119317.s002.doc]

| Component † | SS type | SS subtype* | SS namea | Coreb | HMM/Fastad | Reference |
| --- | --- | --- | --- | --- | --- | --- |
| FlaG | T3SS | Flagellar | - | NO | HMM | This study |
| FlbD | T3SS | Flagellar | - | NO | HMM | This study |
| FleN | T3SS | Flagellar | - | NO | HMM | This study |
| FleS | T3SS | Flagellar | - | NO | HMM | This study |
| FlgA | T3SS | Flagellar | - | NO | HMM | This study |
| FlgB | T3SS | Flagellar | - | YES | HMM | 1 |
| FlgC | T3SS | Flagellar | - | YES | HMM | 1 |
| FlgD | T3SS | Flagellar | - | NO | HMM | 1 |
| FlgE | T3SS | Flagellar | - | NO | HMM | 1 |
| FlgF | T3SS | Flagellar | - | NO | HMM | 1 |
| FlgG | T3SS | Flagellar | - | NO | HMM | 1 |
| FlgH | T3SS | Flagellar | - | NO | HMM | 1 |
| FlgI | T3SS | Flagellar | - | NO | HMM | This study |
| FlgJ | T3SS | Flagellar | - | NO | HMM | This study |
| FlgK | T3SS | Flagellar | - | NO | HMM | 1 |
| FlgL | T3SS | Flagellar | - | NO | HMM | 1 |
| FlgM | T3SS | Flagellar | - | NO | HMM | This study |
| FlgN | T3SS | Flagellar | - | NO | HMM | This study |
| FlhA | T3SS | Flagellar | - | YES | HMM | 1 |
| FlhB | T3SS | Flagellar | - | YES | HMM | 1 |
| FlhF | T3SS | Flagellar | - | NO | HMM | This study |
| FliA | T3SS | Flagellar | - | NO | HMM | This study |
| FliC | T3SS | Flagellar | - | NO | HMM | 1 |
| FliD | T3SS | Flagellar | - | NO | HMM | This study |
| FliE | T3SS | Flagellar | - | YES | HMM | 1 |
| FliF | T3SS | Flagellar | - | YES | HMM | 1 |
| FliG | T3SS | Flagellar | - | NO | HMM | 1 |
| FliH | T3SS | Flagellar | - | NO | HMM | 1 |
| FliI | T3SS | Flagellar | - | NO | HMM | 1 |
| FliJ | T3SS | Flagellar | - | NO | HMM | This study |
| FliK | T3SS | Flagellar | - | NO | HMM | 1 |
| FliL | T3SS | Flagellar | - | YES | HMM | 1 |
| FliM | T3SS | Flagellar | - | YES | HMM | 1 |
| FliN | T3SS | Flagellar | - | YES | HMM | 1 |
| FliO | T3SS | Flagellar | - | NO | HMM | This study |
| FliP | T3SS | Flagellar | - | YES | HMM | 1 |
| FliQ | T3SS | Flagellar | - | YES | HMM | 1 |
| FliR | T3SS | Flagellar | - | YES | HMM | 1 |
| FliS | T3SS | Flagellar | - | NO | HMM | This study |
| FliT | T3SS | Flagellar | - | NO | Fasta | This study |
| FliW | T3SS | Flagellar | - | NO | Fasta | This study |
| FliY | T3SS | Flagellar | - | NO | Fasta | This study |
| FliZ | T3SS | Flagellar | - | NO | Fasta | This study |
| MotA | T3SS | Flagellar | - | NO | HMM | 1 |
| MotB | T3SS | Flagellar | - | NO | HMM | 1 |
| BsaR | T3SS | Non-Flagellar | - | NO | Fasta | This study |
| BsaT | T3SS | Non-Flagellar | - | NO | Fasta | This study |
| BsaU | T3SS | Non-Flagellar | - | NO | Fasta | This study |
| SctC | T3SS | Non-Flagellar | - | YES | HMM | 1 |
| SctD | T3SS | Non-Flagellar | - | NO | HMM | 1 |
| SctF | T3SS | Non-Flagellar | - | NO | HMM | 1 |
| SctI | T3SS | Non-Flagellar | - | NO | HMM | 1 |
| SctJ | T3SS | Non-Flagellar | - | YES | HMM | 1 |
| SctK | T3SS | Non-Flagellar | - | NO | HMM | 1 |
| SctL | T3SS | Non-Flagellar | - | NO | HMM | 1 |
| SctN | T3SS | Non-Flagellar | - | YES | HMM | 1 |
| SctO | T3SS | Non-Flagellar | - | NO | HMM | 1 |
| SctP | T3SS | Non-Flagellar | - | NO | HMM | 1 |
| SctQ | T3SS | Non-Flagellar | - | YES | HMM | 1 |
| SctR | T3SS | Non-Flagellar | - | YES | HMM | 1 |
| SctS | T3SS | Non-Flagellar | - | YES | HMM | 1 |
| SctT | T3SS | Non-Flagellar | - | YES | HMM | 1 |
| SctU | T3SS | Non-Flagellar | - | YES | HMM | 1 |
| SctV | T3SS | Non-Flagellar | - | YES | HMM | 1 |
| SctW | T3SS | Non-Flagellar | - | NO | HMM | 1 |
| TraA_F | T4SS | F | Tra_F | YES | HMM | 2 |
| TraB_F | T4SS | F | Tra_F | YES | HMM | 2 |
| TraC_F | T4SS | F | Tra_F | YES | HMM | 2 |
| TraD_F | T4SS | F | Tra_F | YES | HMM | 2 |
| TraE_F | T4SS | F | Tra_F | YES | HMM | 2 |
| TraF_F | T4SS | F | Tra_F | YES | HMM | 2 |
| TraG_F | T4SS | F | Tra_F | YES | HMM | 2 |
| TraH_F | T4SS | F | Tra_F | YES | HMM | 2 |
| TraI_F | T4SS | F | Tra_F | YES | HMM | 2 |
| TraJ_F | T4SS | F | Tra_F | NO | Fasta | This study |
| TraK_F | T4SS | F | Tra_F | YES | HMM | 2 |
| TraL_F | T4SS | F | Tra_F | YES | HMM | 2 |
| TraM_F | T4SS | F | Tra_F | YES | HMM | 2 |
| TraN_F | T4SS | F | Tra_F | YES | HMM | 2 |
| TraP_F | T4SS | F | Tra_F | NO | Fasta | This study |
| TraQ_F | T4SS | F | Tra_F | YES | HMM | 2 |
| TraR_F | T4SS | F | Tra_F | NO | Fasta | This study |
| TraS_F | T4SS | F | Tra_F | NO | Fasta | This study |
| TraT_F | T4SS | F | Tra_F | NO | Fasta | This study |
| TraU_F | T4SS | F | Tra_F | YES | HMM | 2 |
| TraV_F | T4SS | F | Tra_F | YES | HMM | 2 |
| TraW_F | T4SS | F | Tra_F | YES | HMM | 2 |
| TraX_F | T4SS | F | Tra_F | YES | HMM | 2 |
| TraY_F | T4SS | F | Tra_F | YES | HMM | 2 |
| TrbA_F | T4SS | F | Tra_F | NO | Fasta | This study |
| TrbB_F | T4SS | F | Tra_F | NO | HMM | This study |
| TrbC_F | T4SS | F | Tra_F | YES | HMM | 2 |
| TrbD_F | T4SS | F | Tra_F | NO | Fasta | This study |
| TrbE_F | T4SS | F | Tra_F | NO | Fasta | This study |
| TrbF_F | T4SS | F | Tra_F | NO | Fasta | This study |
| TrbI_F | T4SS | F | Tra_F | NO | Fasta | This study |
| TrbJ_F | T4SS | F | Tra_F | NO | Fasta | This study |
| Orf169_F | T4SS | F | Tra_F | YES | HMM | 2 |
| Tfc1 | T4SS | G | Tfc | YES | Fasta | 2 |
| Tfc2 | T4SS | G | Tfc | YES | HMM | 2 |
| Tfc3 | T4SS | G | Tfc | YES | HMM | 2 |
| Tfc4 | T4SS | G | Tfc | YES | HMM | 2 |
| Tfc5 | T4SS | G | Tfc | YES | HMM | 2 |
| Tfc6 | T4SS | G | Tfc | YES | HMM | 2 |
| Tfc7 | T4SS | G | Tfc | YES | Fasta | 2 |
| Tfc8 | T4SS | G | Tfc | YES | HMM | 2 |
| Tfc9 | T4SS | G | Tfc | YES | HMM | 2 |
| Tfc10 | T4SS | G | Tfc | YES | HMM | 2 |
| Tfc11 | T4SS | G | Tfc | YES | HMM | 2 |
| Tfc12 | T4SS | G | Tfc | YES | HMM | 2 |
| Tfc13 | T4SS | G | Tfc | YES | HMM | 2 |
| Tfc14 | T4SS | G | Tfc | YES | HMM | 2 |
| Tfc15 | T4SS | G | Tfc | YES | HMM | 2 |
| Tfc16 | T4SS | G | Tfc | YES | HMM | 2 |
| Tfc17 | T4SS | G | Tfc | YES | Fasta | 2 |
| Tfc18 | T4SS | G | Tfc | YES | Fasta | 2 |
| Tfc19 | T4SS | G | Tfc | YES | HMM | 2 |
| Tfc20 | T4SS | G | Tfc | YES | Fasta | 2 |
| Tfc21 | T4SS | G | Tfc | YES | Fasta | 2 |
| Tfc22 | T4SS | G | Tfc | YES | HMM | 2 |
| Tfc23 | T4SS | G | Tfc | YES | HMM | 2 |
| Tfc24 | T4SS | G | Tfc | YES | HMM | 2 |
| Orf2_p | T4SS | GP | Orf_p | YES | Fasta | 2 |
| Orf3_p | T4SS | GP | Orf_p | YES | Fasta | 2 |
| Orf5_p | T4SS | GP | Orf_p | YES | Fasta | 2 |
| Orf7_p | T4SS | GP | Orf_p | YES | Fasta | 2 |
| Orf9_p | T4SS | GP | Orf_p | YES | Fasta | 2 |
| Orf10_p | T4SS | GP | Orf_p | YES | Fasta | 2 |
| Orf11_p | T4SS | GP | Orf_p | YES | Fasta | 2 |
| Orf12_p | T4SS | GP | Orf_p | YES | Fasta | 2 |
| Orf13_p | T4SS | GP | Orf_p | YES | Fasta | 2 |
| Orf15_p | T4SS | GP | Orf_p | YES | Fasta | 2 |
| Orf13_Tn | T4SS | GP | Orf_Tn | YES | HMM | 2 |
| Orf14_Tn | T4SS | GP | Orf_Tn | YES | HMM | 2 |
| Orf15_Tn | T4SS | GP | Orf_Tn | YES | HMM | 2 |
| Orf16_Tn | T4SS | GP | Orf_Tn | YES | HMM | 2 |
| Orf17_Tn | T4SS | GP | Orf_Tn | YES | HMM | 2 |
| Orf19_Tn | T4SS | GP | Orf_Tn | YES | HMM | 2 |
| Orf21_Tn | T4SS | GP | Orf_Tn | YES | HMM | 2 |
| PcfC | T4SS | GP | Prg/pcf | YES | Fasta | 2 |
| PcfH | T4SS | GP | Prg/pcf | YES | Fasta | 2 |
| PrgA | T4SS | GP | Prg/pcf | YES | Fasta | 2 |
| PrgB | T4SS | GP | Prg/pcf | YES | Fasta | 2 |
| PrgC | T4SS | GP | Prg/pcf | YES | Fasta | 2 |
| PrgD | T4SS | GP | Prg/pcf | YES | Fasta | 2 |
| PrgF | T4SS | GP | Prg/pcf | YES | Fasta | 2 |
| PrgH | T4SS | GP | Prg/pcf | YES | Fasta | 2 |
| PrgI | T4SS | GP | Prg/pcf | YES | Fasta | 2 |
| PrgJ | T4SS | GP | Prg/pcf | YES | Fasta | 2 |
| PrgK | T4SS | GP | Prg/pcf | YES | Fasta | 2 |
| PrgL | T4SS | GP | Prg/pcf | YES | Fasta | 2 |
| TcpA | T4SS | GP | Tcp | YES | Fasta | 2 |
| TcpC | T4SS | GP | Tcp | YES | Fasta | 2 |
| TcpD | T4SS | GP | Tcp | YES | Fasta | 2 |
| TcpE | T4SS | GP | Tcp | YES | Fasta | 2 |
| TcpF | T4SS | GP | Tcp | YES | Fasta | 2 |
| TcpG | T4SS | GP | Tcp | YES | Fasta | 2 |
| TcpH | T4SS | GP | Tcp | YES | Fasta | 2 |
| YdcQ | T4SS | GP | Ydd | YES | HMM | 2 |
| YddB | T4SS | GP | Ydd | YES | HMM | 2 |
| YddC | T4SS | GP | Ydd | YES | HMM | 2 |
| YddD | T4SS | GP | Ydd | YES | HMM | 2 |
| YddE | T4SS | GP | Ydd | YES | HMM | 2 |
| YddG | T4SS | GP | Ydd | YES | HMM | 2 |
| YddH | T4SS | GP | Ydd | YES | HMM | 2 |
| DotA | T4SS | I | Dot/Icm | YES | Fasta | 2 |
| DotB | T4SS | I | Dot/Icm | YES | Fasta | 2 |
| DotC | T4SS | I | Dot/Icm | YES | Fasta | 2 |
| DotD | T4SS | I | Dot/Icm | YES | Fasta | 2 |
| IcmB | T4SS | I | Dot/Icm | YES | Fasta | 2 |
| IcmC | T4SS | I | Dot/Icm | YES | Fasta | 2 |
| IcmD | T4SS | I | Dot/Icm | YES | Fasta | 2 |
| IcmE | T4SS | I | Dot/Icm | YES | Fasta | 2 |
| IcmF | T4SS | I | Dot/Icm | YES | Fasta | 2 |
| IcmG | T4SS | I | Dot/Icm | YES | Fasta | 2 |
| IcmH | T4SS | I | Dot/Icm | YES | Fasta | 2 |
| IcmJ | T4SS | I | Dot/Icm | YES | Fasta | 2 |
| IcmK | T4SS | I | Dot/Icm | YES | Fasta | 2 |
| IcmL | T4SS | I | Dot/Icm | YES | Fasta | 2 |
| IcmM | T4SS | I | Dot/Icm | YES | Fasta | 2 |
| IcmN | T4SS | I | Dot/Icm | YES | Fasta | 2 |
| IcmO | T4SS | I | Dot/Icm | YES | Fasta | 2 |
| IcmP | T4SS | I | Dot/Icm | YES | Fasta | 2 |
| IcmQ | T4SS | I | Dot/Icm | YES | Fasta | 2 |
| IcmR | T4SS | I | Dot/Icm | YES | Fasta | 2 |
| IcmS | T4SS | I | Dot/Icm | YES | Fasta | 2 |
| IcmT | T4SS | I | Dot/Icm | YES | Fasta | 2 |
| IcmV | T4SS | I | Dot/Icm | YES | Fasta | 2 |
| IcmW | T4SS | I | Dot/Icm | YES | Fasta | 2 |
| IcmX | T4SS | I | Dot/Icm | YES | Fasta | 2 |
| LvgA | T4SS | I | Dot/Icm | YES | HMM | 2 |
| TraH_I | T4SS | I | Tra_I | YES | HMM | 2 |
| TraI_I | T4SS | I | Tra_I | YES | HMM | 2 |
| TraJ_I | T4SS | I | Tra_I | YES | HMM | 2 |
| TraK_I | T4SS | I | Tra_I | YES | HMM | 2 |
| TraL_I | T4SS | I | Tra_I | YES | HMM | 2 |
| TraM_I | T4SS | I | Tra_I | YES | HMM | 2 |
| TraN_I | T4SS | I | Tra_I | YES | HMM | 2 |
| TraO_I | T4SS | I | Tra_I | YES | HMM | 2 |
| TraP_I | T4SS | I | Tra_I | YES | HMM | 2 |
| TraQ_I | T4SS | I | Tra_I | YES | HMM | 2 |
| TraR_I | T4SS | I | Tra_I | YES | HMM | 2 |
| TraS_I | T4SS | I | Tra_I | YES | Fasta | 2 |
| TraT_I | T4SS | I | Tra_I | YES | HMM | 2 |
| TraU_I | T4SS | I | Tra_I | YES | HMM | 2 |
| TraV_I | T4SS | I | Tra_I | YES | Fasta | 2 |
| TraW_I | T4SS | I | Tra_I | YES | HMM | 2 |
| TraX_I | T4SS | I | Tra_I | YES | HMM | 2 |
| TraY_I | T4SS | I | Tra_I | YES | HMM | 2 |
| TrbA_I | T4SS | I | Tra_I | YES | HMM | 2 |
| TrbB_I | T4SS | I | Tra_I | YES | HMM | 2 |
| TrbC_I | T4SS | I | Tra_I | YES | HMM | 2 |
| Cag1 | T4SS | P | Cag | NO | Fasta | This study |
| CagB | T4SS | P | Cag | NO | Fasta | This study |
| CagC | T4SS | P | Cag | YES | Fasta | 2 |
| CagD | T4SS | P | Cag | YES | Fasta | 2 |
| CagE | T4SS | P | Cag | YES | Fasta | 2 |
| CagF | T4SS | P | Cag | YES | Fasta | 2 |
| CagG | T4SS | P | Cag | YES | Fasta | 2 |
| CagH | T4SS | P | Cag | YES | Fasta | 2 |
| CagI | T4SS | P | Cag | YES | Fasta | 2 |
| CagL | T4SS | P | Cag | YES | Fasta | 2 |
| CagM | T4SS | P | Cag | YES | Fasta | 2 |
| CagN | T4SS | P | Cag | YES | Fasta | 2 |
| CagQ | T4SS | P | Cag | NO | Fasta | This study |
| CagS | T4SS | P | Cag | NO | Fasta | This study |
| CagT | T4SS | P | Cag | YES | Fasta | 2 |
| CagU | T4SS | P | Cag | YES | Fasta | 2 |
| CagV | T4SS | P | Cag | YES | Fasta | 2 |
| CagW | T4SS | P | Cag | YES | Fasta | 2 |
| CagX | T4SS | P | Cag | YES | Fasta | 2 |
| CagY | T4SS | P | Cag | YES | Fasta | 2 |
| CagZ | T4SS | P | Cag | YES | Fasta | 2 |
| Cag15 | T4SS | P | Cag | NO | Fasta | This study |
| Cag26 | T4SS | P | Cag | NO | Fasta | This study |
| CagBeta | T4SS | P | Cag | YES | Fasta | 2 |
| CagAlpha | T4SS | P | Cag | YES | Fasta | 2 |
| CagDelta | T4SS | P | Cag | YES | Fasta | 2 |
| CagGamma | T4SS | P | Cag | YES | Fasta | 2 |
| Cjp01 | T4SS | P | Cjp | YES | Fasta | 2 |
| Cjp02 | T4SS | P | Cjp | YES | Fasta | 2 |
| Cjp03 | T4SS | P | Cjp | YES | Fasta | 2 |
| Cjp05 | T4SS | P | Cjp | YES | Fasta | 2 |
| Cjp06 | T4SS | P | Cjp | YES | Fasta | 2 |
| Cjp53 | T4SS | P | Cjp | YES | Fasta | 2 |
| Cjp54 | T4SS | P | Cjp | YES | Fasta | 2 |
| ComB2 | T4SS | P | ComB | YES | HMM | 2 |
| ComB3 | T4SS | P | ComB | YES | HMM | 2 |
| ComB4 | T4SS | P | ComB | YES | HMM | 2 |
| ComB6 | T4SS | P | ComB | YES | HMM | 2 |
| ComB7 | T4SS | P | ComB | YES | HMM | 2 |
| ComB8 | T4SS | P | ComB | YES | HMM | 2 |
| ComB9 | T4SS | P | ComB | YES | HMM | 2 |
| ComB10 | T4SS | P | ComB | YES | Fasta | 2 |
| LvhB2 | T4SS | P | Lvh | YES | Fasta | 2 |
| LvhB3 | T4SS | P | Lvh | YES | Fasta | 2 |
| LvhB4 | T4SS | P | Lvh | YES | Fasta | 2 |
| LvhB5 | T4SS | P | Lvh | YES | Fasta | 2 |
| LvhB6 | T4SS | P | Lvh | YES | Fasta | 2 |
| LvhB7 | T4SS | P | Lvh | YES | Fasta | 2 |
| LvhB8 | T4SS | P | Lvh | YES | Fasta | 2 |
| LvhB9 | T4SS | P | Lvh | YES | Fasta | 2 |
| LvhD4 | T4SS | P | Lvh | YES | Fasta | 2 |
| LvhB10 | T4SS | P | Lvh | YES | Fasta | 2 |
| LvhB11 | T4SS | P | Lvh | YES | Fasta | 2 |
| MagB01 | T4SS | P | MagB | YES | Fasta | 2 |
| MagB02 | T4SS | P | MagB | YES | Fasta | 2 |
| MagB03 | T4SS | P | MagB | YES | Fasta | 2 |
| MagB04 | T4SS | P | MagB | YES | Fasta | 2 |
| MagB05 | T4SS | P | MagB | YES | Fasta | 2 |
| MagB06 | T4SS | P | MagB | YES | Fasta | 2 |
| MagB07 | T4SS | P | MagB | YES | Fasta | 2 |
| MagB08 | T4SS | P | MagB | YES | Fasta | 2 |
| MagB09 | T4SS | P | MagB | YES | Fasta | 2 |
| MagB10 | T4SS | P | MagB | YES | Fasta | 2 |
| MagB11 | T4SS | P | MagB | YES | Fasta | 2 |
| MagB12 | T4SS | P | MagB | YES | Fasta | 2 |
| MagB13 | T4SS | P | MagB | YES | Fasta | 2 |
| MagB14 | T4SS | P | MagB | YES | Fasta | 2 |
| PtlA | T4SS | P | Ptl | YES | Fasta | 2 |
| PtlB | T4SS | P | Ptl | YES | Fasta | 2 |
| PtlC | T4SS | P | Ptl | YES | Fasta | 2 |
| PtlD | T4SS | P | Ptl | YES | Fasta | 2 |
| PtlE | T4SS | P | Ptl | YES | Fasta | 2 |
| PtlF | T4SS | P | Ptl | YES | Fasta | 2 |
| PtlG | T4SS | P | Ptl | YES | Fasta | 2 |
| PtlH | T4SS | P | Ptl | YES | Fasta | 2 |
| PtlI | T4SS | P | Ptl | YES | Fasta | 2 |
| TraB | T4SS | P | Trb | NO | HMM | 3 |
| TraC | T4SS | P | Trb | NO | HMM | 3 |
| TraD | T4SS | P | Trb | NO | HMM | 3 |
| TraF | T4SS | P | Trb | YES | HMM | 2 |
| TraG | T4SS | P | Trb | YES | HMM | 2 |
| TraI | T4SS | P | Trb | NO | HMM | 3 |
| TraM | T4SS | P | Trb | NO | Fasta | 3 |
| TrbB | T4SS | P | Trb | YES | HMM | 2 |
| TrbC | T4SS | P | Trb | YES | HMM | 2 |
| TrbD | T4SS | P | Trb | YES | HMM | 2 |
| TrbE | T4SS | P | Trb | YES | HMM | 2 |
| TrbF | T4SS | P | Trb | YES | HMM | 2 |
| TrbG | T4SS | P | Trb | YES | HMM | 2 |
| TrbH | T4SS | P | Trb | YES | HMM | 2 |
| TrbI | T4SS | P | Trb | YES | HMM | 2 |
| TrbJ | T4SS | P | Trb | YES | HMM | 2 |
| TrbK | T4SS | P | Trb | YES | HMM | 2 |
| TrbL | T4SS | P | Trb | YES | HMM | 2 |
| TrbN | T4SS | P | Trb | YES | HMM | 2 |
| TraD_2 | T4SS | P | Trb | NO | Fasta | 3 |
| TraH_2 | T4SS | P | Trb | NO | HMM | 3 |
| TraH_IncP_1 | T4SS | P | Trb | NO | HMM | 3 |
| TraI_IncP_1 | T4SS | P | Trb | NO | HMM | 3 |
| TraJ_IncP_1 | T4SS | P | Trb | NO | HMM | 3 |
| TraK_IncP_1 | T4SS | P | Trb | NO | HMM | 3 |
| TraL_IncP_1 | T4SS | P | Trb | NO | HMM | 3 |
| TraM_IncP_1 | T4SS | P | Trb | NO | HMM | 3 |
| TraO_IncP_1 | T4SS | P | Trb | NO | HMM | 3 |
| TrbH_IncP_1 | T4SS | P | Trb | YES | HMM | 3 |
| TrbM_IncP_1 | T4SS | P | Trb | NO | HMM | 3 |
| TrbN_IncP_1 | T4SS | P | Trb | YES | HMM | 3 |
| TrbO_IncP_1 | T4SS | P | Trb | NO | HMM | 3 |
| TrbP_IncP_1 | T4SS | P | Trb | NO | HMM | 3 |
| TraA_AT_type | T4SS | P | Trb | NO | HMM | 3 |
| TraA_Ti_type | T4SS | P | Trb | NO | HMM | 3 |
| TrwB | T4SS | P | Trw | YES | HMM | 2 |
| TrwD | T4SS | P | Trw | YES | HMM | 2 |
| TrwE | T4SS | P | Trw | YES | HMM | 2 |
| TrwF | T4SS | P | Trw | YES | HMM | 2 |
| TrwG | T4SS | P | Trw | YES | HMM | 2 |
| TrwH | T4SS | P | Trw | YES | HMM | 2 |
| TrwI | T4SS | P | Trw | YES | HMM | 2 |
| TrwJ | T4SS | P | Trw | YES | HMM | 2 |
| TrwK | T4SS | P | Trw | YES | HMM | 2 |
| TrwL | T4SS | P | Trw | YES | HMM | 2 |
| TrwM | T4SS | P | Trw | YES | HMM | 2 |
| TrwN | T4SS | P | Trw | YES | HMM | 2 |
| VirB1 | T4SS | P | VirB | YES | HMM | 2 |
| VirB2 | T4SS | P | VirB | YES | HMM | 2 |
| VirB3 | T4SS | P | VirB | YES | HMM | 2 |
| VirB4 | T4SS | P | VirB | YES | HMM | 2 |
| VirB5 | T4SS | P | VirB | YES | HMM | 2 |
| VirB6 | T4SS | P | VirB | YES | HMM | 2 |
| VirB7 | T4SS | P | VirB | YES | HMM | 2 |
| VirB8 | T4SS | P | VirB | YES | HMM | 2 |
| VirB9 | T4SS | P | VirB | YES | HMM | 2 |
| VirC1 | T4SS | P | VirB | NO | HMM | 3 |
| VirC2 | T4SS | P | VirB | NO | HMM | 3 |
| VirD1 | T4SS | P | VirB | NO | HMM | 3 |
| VirD2 | T4SS | P | VirB | NO | HMM | 3 |
| VirD4 | T4SS | P | VirB | YES | HMM | 2 |
| VirB10 | T4SS | P | VirB | YES | HMM | 2 |
| VirB11 | T4SS | P | VirB | YES | HMM | 2 |
| ClpB | T6SS | - | - | YES | HMM | 4 |
| ClpV | T6SS | - | - | NO | HMM | 4 |
| HcP | T6SS | - | - | YES | HMM | 4 |
| ImpM | T6SS | - | - | NO | Fasta | This study |
| VasA | T6SS | - | - | YES | HMM | 4 |
| VasF | T6SS | - | - | YES | HMM | 4 |
| VasH | T6SS | - | - | YES | HMM | 4 |
| VasK | T6SS | - | - | YES | HMM | 4 |
| VCA0107 | T6SS | - | - | YES | HMM | 4 |
| VCA0108 | T6SS | - | - | YES | HMM | 4 |
| VCA0109 | T6SS | - | - | NO | HMM | 4 |
| VCA0111 | T6SS | - | - | YES | HMM | 4 |
| VCA0112 | T6SS | - | - | YES | HMM | 4 |
| VCA0113 | T6SS | - | - | YES | HMM | 4 |
| VCA0114 | T6SS | - | - | YES | HMM | 4 |
| VCA0118 | T6SS | - | - | NO | HMM | 4 |
| VCA0119 | T6SS | - | - | NO | HMM | 4 |
| VCA0121 | T6SS | - | - | NO | HMM | 4 |
| VCA0122 | T6SS | - | - | NO | Fasta | 4 |
| VgrG | T6SS | - | - | YES | HMM | 4 |

 Orthologous clusters, which correspond to table entries with Reference field value of 3, are usually composed by more than one component.

† Components of the T6SS have been named after those of Vibrio (4).

* F, P, I, G and GP subtypes correspond to T4SS types of Classification Scheme 2 used in Bi et al., 2012 (2) for T4SS.

a Nomenclature of T4SS names as used in Bi et al., 2012 (2). 

b Whether a given component belongs to the core set of components described for the type of secretion system it is part of. See main text for more details.

d Whether the sequence information of a given component is stored in the database as a HMM profile or as a multi-FASTA file.

References

1 Abby SS, Rocha EPC (2012) The Non-Flagellar Type III Secretion System Evolved from the Bacterial Flagellum and Diversified into Host-Cell Adapted Systems. PLoS Genetics 8: e1002983.

2 Bi D, Liu L, Tai C, Deng Z, Rajakumar K, et al. (2012) SecReT4: a web-based bacterial type IV secretion system resource. Nucleic Acids Res 41: D660-5.

3 Souza RC, Quispe Saji GD, Costa MO, Netto DS, Lima NC, et al. (2012) AtlasT4SS: a curated database for type IV secretion systems. BMC microbiology 12: 172.

4 Shrivastava S, Mande SS (2008) Identification and functional characterization of gene components of type VI secretion system in bacterial genomes. PLoS One 3(8): e2955.
